# Copy-Number Aberrations in Circulating Tumor DNA Enable Diagnosis and Risk Stratification of Pediatric Neuroblastic Tumors

**DOI:** 10.1158/2767-9764.CRC-25-0452

**Published:** 2026-01-06

**Authors:** Ting Tao, Jiabin Cai, Yinbing Tang, Dongfang Lu, Lifeng Zhang, Jinkai Peng, Yilong Wang, Weizhong Gu, Shouhua Zhang, Jinhu Wang

**Affiliations:** 1 Pediatric Cancer Research Center, National Clinical Research Center for Child Health, Children’s Hospital Zhejiang University School of Medicine, Hangzhou, China.; 2 Department of Surgical Oncology, Children’s Hospital Zhejiang University School of Medicine, National Clinical Research Center for Child Health, Hangzhou, China.; 3 Key Laboratory of Diagnosis and Treatment of Neonatal Diseases of Zhejiang Province, Hangzhou, China.; 4 Cancer Center, Zhejiang University, Hangzhou, China.; 5 Department of Neurology, Children’s Hospital Zhejiang University School of Medicine, National Clinical Research Center for Child Health, Hangzhou, China.; 6 Department of Pathology, Children’s Hospital Zhejiang University School of Medicine, National Clinical Research Center for Child Health, Hangzhou, China.; 7 Department of General Surgery, Jiangxi Provincial Children’s Hospital, The Affiliated Children’s Hospital of Nanchang Medical College, Nanchang, China.

## Abstract

Low-pass whole-genome sequencing (LP-WGS) of circulating tumor DNA (ctDNA) is increasingly recognized for its utility in identifying somatic copy-number aberration (CNA). In this study, we analyzed LP-WGS ctDNA data from 73 pediatric patients with neuroblastic tumor and 11 healthy controls to explore diagnostic value of ctDNA CNA burden (including the genotypings) with a customized bioinformatics workflow. We found that a high baseline ctDNA CNA burden [tumor DNA fraction (TFx) ≥0.2%] was present in 36 of 41 patients (87.80%) with neuroblastoma, six of 22 patients (27.27%) with ganglioneuroblastoma, and three of 10 patients (30%) with ganglioneuroma. High baseline ctDNA CNA burden could predict high-risk neuroblastic tumors with an area under curve (AUC) of 0.95, sensitivity of 94.12%, and specificity of 100%. Frequent chromosomal copy-number changes, including chr17q gain, chr7 gain, chr3p loss, and chr11q loss, were found in ctDNA. Gain of chr17q demonstrated the highest diagnostic value with an AUC of 0.92, indicating strong sensitivity and specificity for detecting high-risk neuroblastic tumors. The homologous recombination deficiency score in the high- and intermediate-risk groups was significantly elevated compared with those in the low-/very low–risk group. The TFx levels and segmental alterations significantly decreased in patients with neuroblastic tumor who underwent chemotherapy, from median TFx = 13.82% before treatment to 0.24% after treatment (*P* < 0.0001). Our findings highlight the effectiveness of LP-WGS ctDNA CNA analysis as a promising approach for diagnosis and risk stratification of pediatric neuroblastic tumors and for monitoring chemotherapy response. Particularly, ctDNA analysis is minimally invasive, rapid, and cost-effective, which could bring additional benefits in pediatric practices.

**Significance:**

Our results support the development of ctDNA CNA analysis as a robust and minimally invasive approach for early detection, molecular diagnosis, and risk stratification of peripheral neuroblastic tumors.

## Introduction

Peripheral neuroblastic tumors (pNT) are the most common extracranial solid tumors of childhood that arise from the primitive neuroblasts of the peripheral sympathetic nervous system, typically found in the adrenal glands or paraspinal ganglia (1). According to the International Neuroblastoma Pathology Classification System, pNTs are classified into several basic categories and their subtypes: neuroblastoma (undifferentiated, poorly differentiated, and differentiating), ganglioneuroblastoma (intermixed and nodular), and ganglioneuroma (maturing and mature; refs. 2, 3). Neuroblastoma is the most common and malignant category of these tumors, and *MYCN* gene amplification is found in ∼25% of cases and associated with poor disease prognosis. Ganglioneuroma is the most benign form, comprised of differentiated ganglion cells and mature stroma. Ganglioneuroblastoma contains a mix of mature ganglion cells and immature neuroblasts. It generally has a better prognosis than neuroblastoma, depending on the proportion of immature cells (4, 5). The overall incidence rate of these tumors is approximately 10.2 cases per million children under 14 years and accounts for about 8% to 10% of all childhood cancers and nearly 15% of cancer-related deaths in children (1, 6, 7). Once pNTs are diagnosed, staging is essential for guiding treatment and prognosis. The International Neuroblastoma Staging System (INSS) is used to stage these tumors based on surgical findings and tumor spread, categorizing them into INSS stage 1, 2A, 2B, 3, 4, or 4S. By using the International Neuroblastoma Risk Group (INRG) staging system and other key prognostic factors (age, histology, *MYCN*, ploidy, and 11q status), the INRG classification system stratifies patients into very low–risk, low-risk, intermediate-risk, or high-risk groups to guide treatment decisions and improve prognosis (1, 8). Although patients with low- and intermediate-risk pNTs generally achieve favorable outcomes (∼80% to 95% event-free survival rate), the high-risk patients still have <50% event-free survival rate (5).

pNTs are diagnosed using a combination of radiographic imaging, biopsy, genetic testing, and biochemical markers. Early detection of pNTs is crucial for effective intervention and improving survival rates. However, pNTs lack distinctive early symptoms, leading to challenges in early diagnosis. At the time of diagnosis, approximately 50% of patients present with localized disease, whereas approximately 35% have already developed regional lymph node involvement (9). To aid early detection, Japan launched a mass screening for pNTs by measurement of catecholamine metabolites in urine in the 1970s, followed by Canada and Germany (10–12). These studies demonstrated that screening for pNTs by urinary catecholamine metabolites increased the disease incidence but led to substantial overdiagnosis of nonthreatening cases, without decreasing the incidence of advanced-stage disease and mortality rates (13). Liquid biopsy, which analyzes circulating tumor cells (CTC) and circulating cell-free tumor DNA (ctDNA) in the blood of patients with cancer, is increasingly recognized for its diagnostic and monitoring potential. CTCs can offer insights into metastatic potential, whereas ctDNA reflects genetic alterations present in tumor cells, providing real-time information on tumor burden, treatment response, and genetic evolution (14). Early studies of ctDNA in pNTs focused on the detection of *MYCN* copy number as a specific biomarker for neuroblastoma, offering insights into disease aggressiveness (15–27). Additionally, the copy number and pathogenic variations, including but not limited to those involving ALK, have been explored as complementary biomarkers (21–25, 28, 29). In particular, by digital droplet PCR targeting ALK and serial ctDNA sequencing, scientists can define clinically relevant clonal evolution in response to ALK inhibition therapy in patients with neuroblastoma (23, 30–32). Other biomarkers, including methylated DCR2 and RASSF1A, 5-hydroxymethylcytosine, and 17q gain and 11p loss, have been identified in ctDNA of patients with neuroblastoma (33–39). These findings represent a promising minimally invasive approach to detect and monitor specific genetic alterations associated with neuroblastoma. Further studies showed that the plasma ctDNA concentration was highly correlated to neuroblastoma tumor burden and dynamically altered in response to chemotherapy (40–44). Chicard and colleagues and Van Roy and colleagues showed that copy-number aberration (CNA) profiles in ctDNA were highly concordant with those from primary tumor tissues in patients with neuroblastoma, demonstrating the feasibility of ctDNA CNA in predicting tumor DNA copy-number profiles (45–48). Although these studies highlight the feasibility of using ctDNA to capture genetic alterations and CNA to reflect tumor burden, the clinical utility of ctDNA in guiding diagnosis and risk stratification of pNTs remains to be fully validated.

In this study, we employed low-pass whole-genome sequencing (LP-WGS) coupled with a customized bioinformatics workflow, named ultrasensitive chromosomal aneuploidy detection of ctDNA in pNTs (pNT-UCAD), to explore the clinical relevance of ctDNA CNA burden (including the genotypings) in pNTs. We demonstrated the effectiveness of LP-WGS ctDNA CNA analysis as a promising approach for diagnosis and risk stratification of pediatric neuroblastic tumors and for monitoring chemotherapy response. Particularly, ctDNA analysis is minimally invasive, rapid, and cost-effective, which could bring additional benefits in pediatric practices.

## Materials and Methods

### Patient characteristics and ethical statement

Seventy-three plasma samples were obtained from patients with pNT (*n* = 73) prior to chemotherapy, whereas 16 were collected after chemotherapy and were paired with their corresponding prechemotherapy samples. The demographic characteristics of these patients are presented as Supplementary Table S1. Histopathologic features of tumors were evaluated by the Department of Pathology, Children’s Hospital Zhejiang University School of Medicine. In addition, 11 plasma samples from healthy controls (*n* = 11; male:female = 8:3, median age = 4 years, and age range = 1–13 years) were obtained. All results were reported with arbitrary sample ID numbers without linked identifiers. This study was conducted in accordance with the Declaration of Helsinki and approved by the Ethics Committee of Children’s Hospital, Zhejiang University School of Medicine (2020-IRB-049). Data were acquired with written informed consent from patients, their parents, or guardians.

### LP-WGS

ctDNA was extracted from plasma samples by using the QIAseq cfDNA Extraction kit (Qiagen). For LP-WGS, 10 ng ctDNA was used to prepare the sequencing libraries with the NEBNext Ultra II FS DNA Library Prep Kit (NEB). DNA fragments were ligated with eight base-barcoded sequencing adapters and amplified by PCR. Purified sequencing libraries were massively sequenced by the Illumina HiSeq X Ten platform (Illumina) at Suzhou Hongyuan Biological Technology Co., Ltd. with a 150-base paired-end reads strategy, providing efficient and high-throughput low-pass sequencing. Segmental copy numbers were determined using a bespoke analytic pipeline, the pNT-UCAD, enabling precise detection of chromosomal copy-number variations.

### Statistical analyses and data visualization

An average of 19.5 million paired-end reads were generated for each sample and subsequently aligned to the human reference genome (hg19) with BWA (RRID: SCR_010910; ref. 49), and genomic coverage was assessed using the samtools mpileup command-line tool (RRID: SCR_002105; ref. 50). The average coverage for each 200-kb bin was calculated, and *Z*-scores for each bin were normalized using the following formula:

$$Z_{bin}={coverage}_{normalized}=\;\frac{{coverage}_{raw}-\;mean\;\left({coverage}_{controls,\;raw}\right)}{stdev\;\left({coverage}_{controls,raw}\right)},$$

in which *Z*_*bin*_ represents standardized *Z*-score for a specific genomic bin, *coverage*_*raw*_ is raw coverage of the bin under investigation, *coverage*_*controls,raw*_ is raw coverage from control samples, *mean* (*coverage*_*controls,raw*_) is mean raw coverage value across control samples, and *stdev* (*coverage*_*controls,raw*_) is SD of raw coverage in control samples.

The circular binary segmentation algorithm, implemented in the R package DNAcopy (RRID: SCR_012560; ref. 51), was employed to identify significant genomic breakpoints and define copy-number segments. The ctDNA fraction derived from tumor cells [tumor DNA fraction (TFx)] was estimated by ichroCNA based on CNA burden (52–54). A *P* value threshold of <0.05 was used to determine statistical significance for the segmentations. For categorical variables, the appropriate statistical test, such as the Wilcoxon test, was applied. The sensitivity and specificity of pNT-UCAD were estimated by ROC curves. Homologous recombination deficiency (HRD) score was estimated by using shallowHRDv2 (55) based on the number of large genomic alterations (the number of copy-number breaks between chromosome segments of at least 10 megabases in size). Data were visualized using R.

## Results

### CNA burden in plasma ctDNA enables diagnosis and risk stratification of pNTs

**Figure 1.**
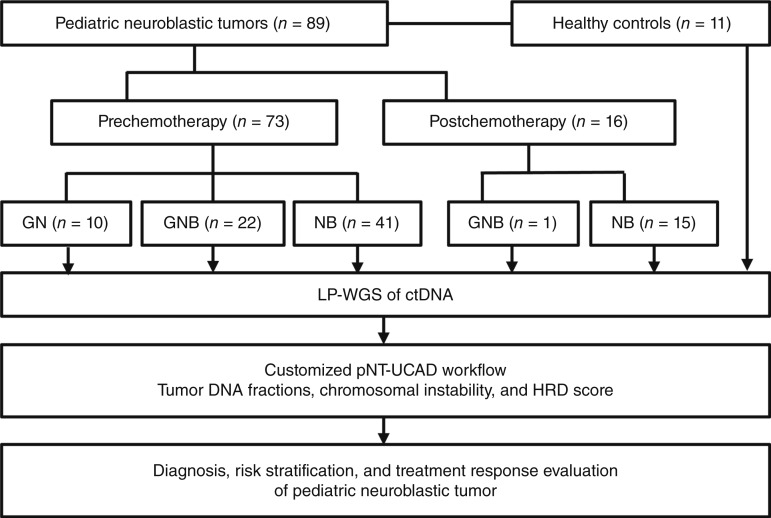
Customized pNT-UCAD workflow designed for diagnosis, risk stratification, and treatment response evaluation of pediatric neuroblastic tumor. GN, ganglioneuroma; GNB, ganglioneuroblastoma; NB, neuroblastoma.

To investigate the clinical utility of ctDNA in guiding diagnosis and risk stratification of pNTs, we enrolled 73 patients with pNT and 11 healthy controls from Children’s Hospital Zhejiang University School of Medicine. The demographic characteristics of these patients are presented as Supplementary Table S1. For patients with pNT, 73 plasma samples were collected before chemotherapy, and 16 postchemotherapy samples were paired with their corresponding prechemotherapy samples for comparative analysis. All the ctDNA from plasma samples of patients with pNT and healthy controls was subjected to LP-WGS, and TFx was determined based on CNA burden (see “Materials and Methods” for detail; Supplementary Table S2). A detailed flow diagram illustrating the samples and workflow of the study is shown in Fig. 1.

**Figure 2.**
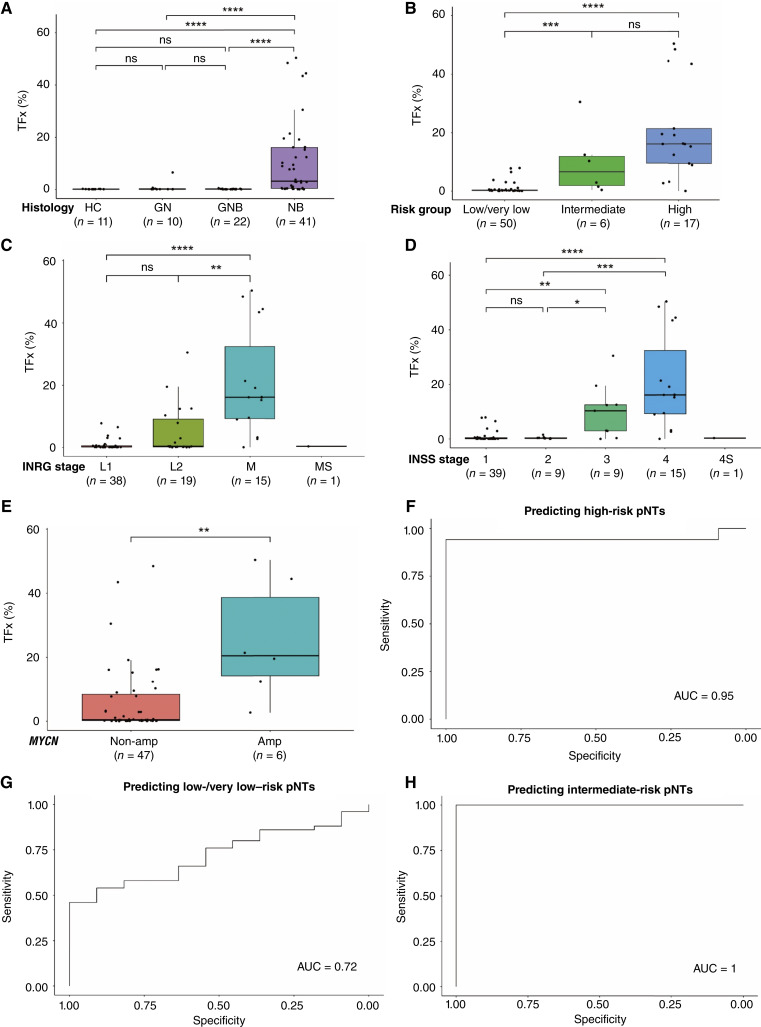
CNA burden in plasma ctDNA enables diagnosis and risk stratification of pNTs. **A–E,** TFx in various clinicopathologic groups of pNTs. Boxes indicate the median (horizontal line), 25th percentile, and 75th percentile; whiskers indicate distances from the largest and smallest value to each end of the box that are within 1.5 × box length. Values were compared with the Wilcoxon test. *, *P* < 0.05; **, *P* < 0.01; ***, *P* < 0.001; ****, *P* < 0.0001; ns, not significant. **F–H,** ROC curves of the pNT-UCAD model for risk-based stratification analysis. The TFx cutoff was defined by maximizing the Youden index (sensitivity + specificity − 1) using the pROC R package. Amp, amplified; GN, ganglioneuroma; GNB, ganglioneuroblastoma; HC, healthy control; NB, neuroblastoma; non-amp, nonamplified.

We began by investigating the diagnostic value of TFx across various categories. The TFx levels in ganglioneuroma (average TFx = 0.79%) and ganglioneuroblastoma (average TFx = 0.15%) were generally low, whereas the neuroblastoma group (average TFx = 10.62%) exhibited significantly higher levels of TFx compared with both other tumor groups (both *P* < 0.0001, Wilcoxon test), as well as the healthy controls (average TFx = 0.099%, *P* < 0.0001, Wilcoxon test; Fig. 2A). Additionally, TFx levels in the intermediate-risk (average TFx = 9.64%) and high-risk (average TFx = 20.43%) groups were significantly elevated compared with those in the low-/very low–risk group (average TFx = 0.83%), respectively (Fig. 2B). For the INRG stage, TFx levels in the M stage were significantly higher than those in the L1 and L2 stages (Fig. 2C). Similarly, TFx levels in the advanced INSS stages (stages 3 and 4) were significantly elevated compared with those in stages 1 and 2 (Fig. 2D). With regard to *MYCN* status, we observed significantly higher levels of TFx in the *MYCN*-amplified group compared with the nonamplified group (Fig. 2E), consisting with the fact that *MYCN*-amplified neuroblastoma typically exhibits more aggressive tumor behavior and higher tumor burden. Taken together, TFx levels are significantly correlated with histology, INRG classification, INRG and INSS stages and *MYCN* status in pNTs (Table 1).

**Table 1.** Statistical analysis of TFx between various clinicopathologic groups in pNTs.

Category	Group 1 (average TFx %, *n*)	Group 2 (average TFx %, *n*)	*P* (Wilcoxon test)	Significance
Histology	HC (0.099, 11)	GN (0.79, 10)	0.699	ns
	HC (0.099, 11)	GNB (0.15, 22)	0.223	ns
	HC (0.099, 11)	NB (10.62, 41)	<0.0001	****
	GN (0.79, 10)	GNB (0.15, 22)	0.611	ns
	GN (0.79, 10)	NB (10.62, 41)	<0.0001	****
	GNB (0.15, 22)	NB (10.62, 41)	<0.0001	****
Risk group^a^	Low/very low (0.83, 50)	Intermediate (9.64, 6)	<0.001	***
	Low/very low (0.83, 50)	High (20.43, 17)	<0.0001	****
	Intermediate (9.64, 6)	High (20.43, 17)	0.101	ns
INRG stage	L1 (0.83, 38)	L2 (5.22, 19)	0.0768	ns
	L1 (0.83, 38)	M (21.02, 15)	<0.0001	****
	L2 (5.22, 19)	M (21.02, 15)	0.00104	**
INSS stage	1 (0.92, 39)	2 (0.41, 9)	0.348	ns
	1 (0.92, 39)	3 (10.13, 9)	0.00132	**
	1 (0.92, 39)	4 (21.02, 15)	<0.0001	****
	2 (0.41, 9)	3 (10.13, 9)	0.0142	*
	2 (0.41, 9)	4 (21.02, 15)	<0.001	***
	3 (10.13, 9)	4 (21.02, 15)	0.123	ns
*MYCN* status	Non-amp (6, 47)	Amp (25.14, 6)	0.00115	**

Abbreviations: Amp, amplified; GN, ganglioneuroma; GNB, ganglioneuroblastoma; HC, healthy control; NB, neuroblastoma; non-amp, nonamplified; ns, not significant.

*, *P* < 0.05; **, *P* < 0.01; ***, *P* < 0.001; ****, *P* < 0.0001.

a Based on INRG classification system.

The diagnostic performance of pNT-UCAD was evaluated based on TFx between healthy controls and various clinicopathologic groups. Although the high-risk group demonstrated excellent diagnostic performance with an area under curve (AUC) of 0.95 [95% confidence interval (CI), 84%–100%], sensitivity of 94.12% (95% CI, 82.35%–100%), and specificity of 100% (95% CI, 100%–100%; Fig. 2F; Table 2), the low-/very low–risk group also achieved considerable diagnostic accuracy, achieving an AUC of 0.72 (Fig. 2G; Table 2). As the intermediate-risk group comprised only six samples, its diagnostic performance could not be fully assessed (Fig. 2H; Table 2). In addition, the neuroblastoma group, INRG stage M, INSS stage 4, and *MYCN*-amplified group achieved high AUC values of 0.94, 0.94, 0.94, and 1, respectively (Table 2). These results further validated the effectiveness of TFx as a diagnostic tool, particularly demonstrating its high diagnostic capability in the high-risk, advanced stages, and *MYCN*-amplified or neuroblastoma groups. These data also emphasize the potential of TFx as a clinically relevant biomarker for disease stratification and risk assessment in pNTs.

**Table 2.** The diagnostic performance of pNT-UCAD evaluated based on TFx in pNTs.

Group	AUC (95% CI)	Sensitivity (95% CI)	Specificity (95% CI)	PPV	NPV	Accuracy
High risk^a^	0.95 (0.84–1)	0.94 (0.82–1)	1 (1–1)	1	0.92	0.96
Intermediate risk^a^	1 (1–1)	1 (1–1)	1 (1–1)	1	1	1
Low/very low risk^a^	0.72 (0.59–0.86)	0.46 (0.36–0.86)	1 (0.73–1)	1	0.29	0.56
NB	0.94 (0.88–1)	0.88 (0.80–0.98)	1 (0.91–1)	1	0.69	0.90
GNB	0.61 (0.41–0.80)	0.45 (0.27–0.68)	0.82 (0.55–1)	0.83	0.43	0.58
GN	0.61 (0.35–0.87)	0.30 (0.20–1)	1 (0.27–1)	1	0.61	0.67
INRG stage L1	0.73 (0.58–0.87)	0.47 (0.37–0.89)	1 (0.64–1)	1	0.35	0.59
INRG stage L2	0.81 (0.65–0.97)	0.74 (0.53–0.95)	0.91 (0.82–1)	0.93	0.67	0.80
INRG stage M	0.94 (0.82–1)	0.93 (0.80–1)	1 (1–1)	1	0.92	0.96
INSS stage 1	0.70 (0.55–0.85)	0.41 (0.31–0.90)	1 (0.55–1)	1	0.32	0.54
INSS stage 2	0.86 (0.67–1)	0.78 (0.44–1)	0.91 (0.73–1)	0.88	0.83	0.85
INSS stage 3	0.89 (0.67–1)	0.89 (0.67–1)	1 (1–1)	1	0.92	0.95
INSS stage 4	0.94 (0.82–1)	0.93 (0.80–1)	1 (1–1)	1	0.92	0.96
*MYCN* amp	1 (1–1)	1 (1–1)	1 (1–1)	1	1	1
*MYCN* non-amp	0.81 (0.70–0.91)	0.68 (0.57–0.85)	1 (0.91–1)	1	0.42	0.74

Abbreviations: amp, amplified; GN, ganglioneuroma; GNB, ganglioneuroblastoma; NB, neuroblastoma; non-amp, non-amplified; NPV, negative predictive value; PPV, positive predictive value.

a Based on INRG classification system.

### Chromosomal instability in plasma ctDNA predicts high-risk group in patients with pNT

**Figure 3.**
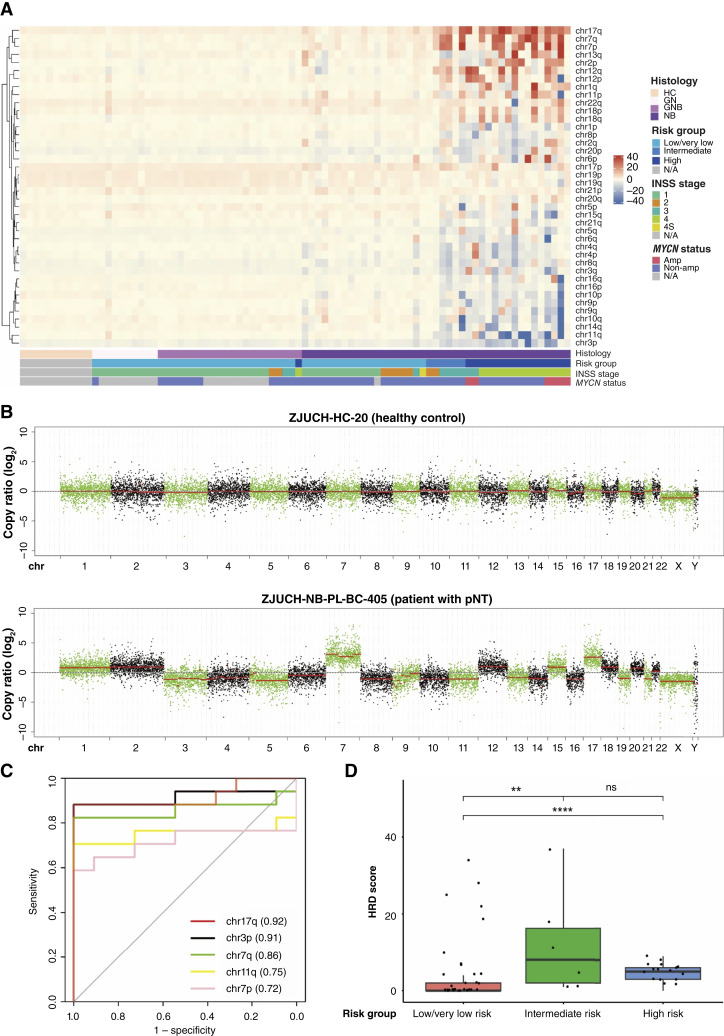
CIN in plasma ctDNA predicts the high-risk group in patients with pNT. **A,** A Heatmap of segmental alterations in ctDNA from patients with pNT. Patient information is shown at the bottom of the panel. Scale bar represents copy-number *Z*-score of each segment. **B,** Representative genome-wide copy-number variation in a healthy child (top) and a patient with pNT (bottom). **C,** ROC curves illustrated the diagnostic performance of chromosomal aberrations in distinguishing between healthy controls and high-risk pNTs. AUC is shown for each chromosomal aberration. **D,** HRD score in different risk groups of pNTs. Boxes indicate the median (horizontal line), 25^th^ percentile, and 75^th^ percentile; whiskers indicate distances from the largest and smallest value to each end of the box that are within 1.5 × box length. Values were compared with the Wilcoxon test. **, *P* < 0.01; ****, *P* < 0.0001; ns, not significant. Amp, amplified; GN, ganglioneuroma; GNB, ganglioneuroblastoma; HC, healthy control; NB, neuroblastoma; non-amp, nonamplified.

To identify common chromosomal aberrations in ctDNA and their clinical relevance for patients with pNT, we grouped the copy-number *Z*-score (Supplementary Table S3) based on clinicopathologic characters. No significant segmental alterations were found in healthy controls [Fig. 3A and B (top)]. By contrast, a number of common chromosomal aberrations of ctDNA in patients with pNT were identified, including gains in chr17q, chr7, chr13q, chr2p, chr12, and chr1q and losses in chr3p, chr11q, chr14q, chr10, chr9, and chr1p [Fig. 3A and B (bottom); Supplementary Table S2]. These aberrations were consistent with the reported segmental alterations in neuroblastoma tumor tissue (1). More importantly, these chromosomal aberrations were enriched in the ctDNA of patients with high-risk or advanced-stage (stages 3 and 4) neuroblastoma (Fig. 3A), indicating their potential association with tumor progression and aggressive clinical behavior.

The ROC curves illustrated the diagnostic performance of chromosomal aberrations in distinguishing between healthy controls and high-risk pNTs (Fig. 3C). Among the most gained or lost chromosomes, gain of chr17q demonstrated the highest diagnostic value with an AUC of 0.92, indicating strong sensitivity (88.24%) and specificity (100%) for detecting high-risk pNTs. Additionally, loss of chr3p and gain of chr7q achieved high AUC values of 0.91 and 0.86, respectively. These results suggest that chromosomal instability (CIN) in chr17q, chr3p, and chr7q may play a pivotal role in neuroblastic tumor progression and can serve as potential biomarkers for diagnosis.

It has been reported that HRD is strongly correlated with CIN in epithelial ovarian cancer, serving as a therapeutic vulnerability to PARP inhibitor (PARPi) or platinum-based neoadjuvant chemotherapy (56). Here, we inferred the homologous recombination status on ctDNA by HRD score for each patient with pNT. The HRD scores in the intermediate-risk (median HRD score = 8) and high-risk (median HRD score = 5) groups were significantly higher compared with those in the low-/very low–risk group (median HRD score = 0), respectively (Fig. 3D). Patients with pNT with high HRD scores may thus potentially benefit from PARPi or platinum-based neoadjuvant chemotherapy. In summary, our analysis suggests that the widespread presence of CIN and copy-number alterations in specific chromosomal regions of ctDNA may represent key molecular features in patients with high-risk pNTs, thus serving as potential diagnostic markers.

### Chemotherapy effectively reduces CNA burden in patients with pNT

**Figure 4.**
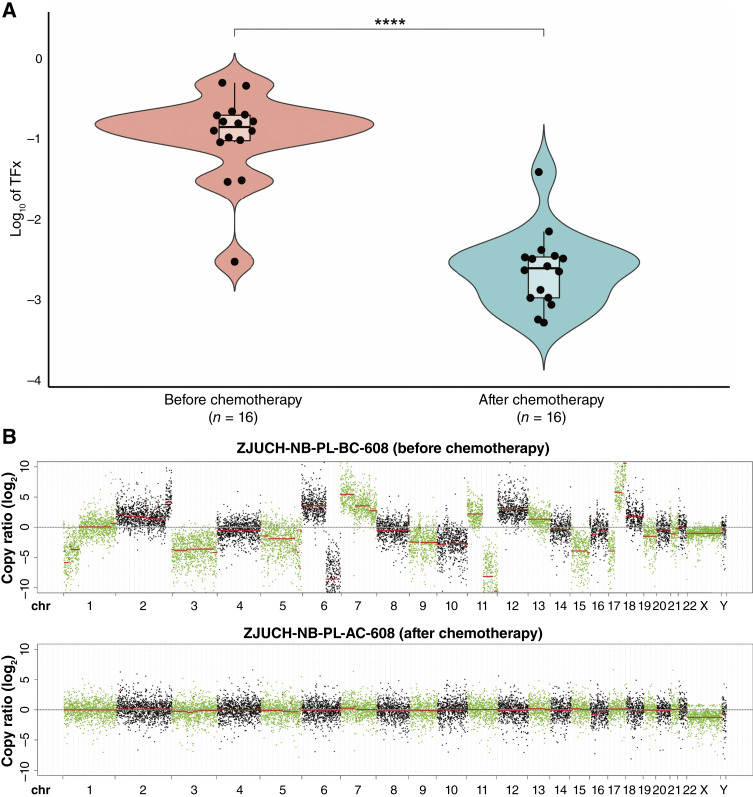
Chemotherapy effectively reduces CNA burden in patients with pNT. **A,** Log_10_ of TFx values in 16 paired ctDNA samples collected before and after chemotherapy from the same patients. Boxes indicate the median (horizontal line), 25th percentile, and 75th percentile; whiskers indicate distances from the largest and smallest value to each end of the box that are within 1.5 × box length. Values were compared with the Wilcoxon test. ****, *P* < 0.0001. **B,** Representative genome-wide copy-number variation in patients with pNT before (top) and after (bottom) chemotherapy.

We next asked whether the CNA burden in ctDNA can predict treatment response in patients with pNT. Sixteen paired ctDNA samples collected before and after chemotherapy from the same patients were subjected to analysis. The TFx levels in the prechemotherapy group ranged from 0.29% to 48.45%, with a median of 13.82% [Fig. 4A and B (top)]. By contrast, the TFx levels in the postchemotherapy group ranged from 0.051% to 3.82%, with a median of 0.24% [Fig. 4A and B (bottom)]. In addition, the number of segmental chromosome alterations was largely reduced after chemotherapy (Fig. 4B; Supplementary Table S2). It is evident that chemotherapy significantly reduced the TFx values and segmental alterations of the samples (*P* < 0.0001, Wilcoxon test). The decrease in TFx and segmental alterations reflects the effectiveness of chemotherapy in eliminating tumor cells. Before treatment, the wide range of TFx levels was likely correlated with high heterogeneity of tumor burden among individuals. After treatment, the significant reduction and narrowing of the TFx range demonstrated the robust capacity of chemotherapy not only to suppress tumor cells but also to potentially eliminate minimal residual disease. Taken together, these data indicate that TFx may serve as a valuable tool for monitoring the effectiveness of chemotherapy and detecting minimal residual disease.

## Discussion

Analysis of ctDNA in blood samples is an emerging area in precision oncology, enabling more dynamic and individualized treatment strategies for patients with cancer (57). ctDNA is detectable in pediatric patients with many common solid tumors, including neuroblastoma, rhabdomyosarcoma, osteosarcoma, and Wilms tumor (58). Its presence provides a potential way to analyze tumor genetics, monitor disease progression, and assess treatment response. ctDNA is typically released into the bloodstream by apoptotic or necrotic tumor cells (57). Previous studies have shown that blood-based ctDNA content was 5.2-fold higher in patients with neuroblastoma compared with pediatric controls (40), indicating that tumor cells are a primary source of ctDNA, reflecting tumor burden. Additionally, the concordance of CNA profiles and genetic alterations between ctDNA and primary tumor tissues in patients with neuroblastoma (45–48, 57, 59) further highlights the potential of ctDNA for noninvasive tumor profiling and monitoring. Early diagnosis of pNTs remains challenging because of their heterogeneous nature and nonspecific early symptoms, and advances in molecular diagnostics and biomarker research hold promise for improving detection and outcomes. By LP-WGS of ctDNA, we established the clinical utility of ctDNA in guiding diagnosis and risk stratification of pNTs. CNA burden in plasma ctDNA reflected by TFx was significantly correlated with histology, risk group, INRG and INSS stages, and *MYCN* status in pNTs. The chromosomal aberrations were enriched in the ctDNA of patients with high-risk or advanced-stage (stages 3 and 4) neuroblastoma. Both the TFx and chromosomal aberrations (chr17q, chr 3p, and chr7q) demonstrated high diagnostic capabilities in the high-risk group of pNTs, providing valuable diagnostic markers. Furthermore, CNA burden in ctDNA could predict treatment response in patients with pNT, enabling dynamic monitoring and real-time assessment of disease progression.

**Figure 5.**
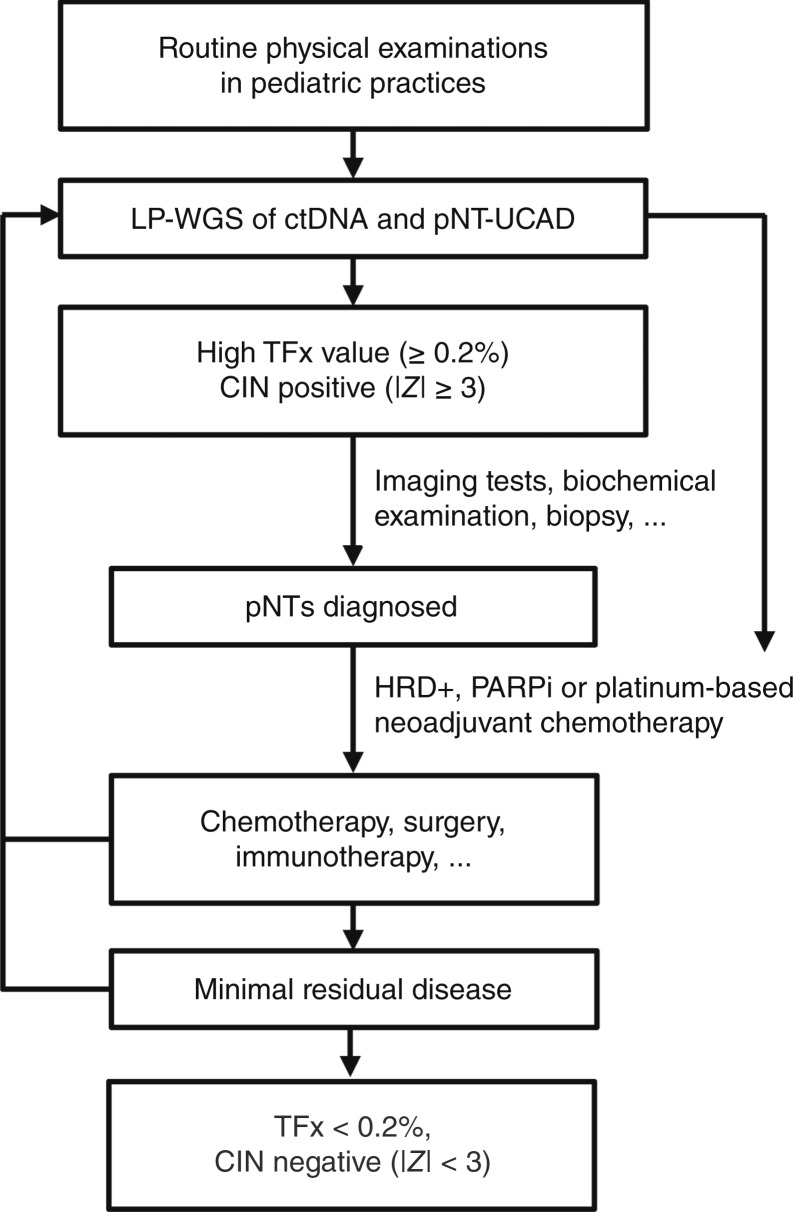
Proposed pNT diagnosis and tumor burden monitoring procedure. Peripheral blood samples are collected during the routine pediatric exams, with ctDNA extracted for LP-WGS and pNT-UCAD analysis. Patients with high TFx values (≥0.2%) and chromosomal aberrations (|*Z*| ≥ 3) are prompted for further evaluation, such as imaging, biochemical tests, and biopsy. Once diagnosed, ctDNA is served as a minimally invasive tool to monitor treatment efficacy and detect minimal residual disease throughout the entire course of therapy, with treatment strategies adjusted based on ctDNA findings. The treatment will be discontinued when patients reach TFx <0.2% and copy number |*Z*| < 3.

Based on these results, we propose a diagnosis and tumor burden monitoring procedure for pNTs (Fig. 5). Peripheral blood samples are collected during the routine physical examinations in pediatric practices, and ctDNA samples are extracted and subjected to LP-WGS. Data are processed with pNT-UCAD to calculate TFx values and identify chromosomal aberrations. Patients with high TFx values (≥0.2%) and chromosomal aberrations (|*Z*| ≥ 3) are prompted for further evaluation, such as imaging tests, biochemical examination, and biopsy. Once diagnosed, ctDNA will continue to serve as a minimally invasive tool to dynamically monitor the effectiveness of treatment and detect minimal residual disease throughout the entire course of therapy. Treatment strategies will be adjusted based on ctDNA findings, such as applying PARPi or platinum-based neoadjuvant chemotherapy for HRD-positive patients, thereby enabling personalized and responsive therapeutic interventions. By integrating physical examinations, advanced ctDNA diagnostics, and tailored therapeutic strategies, this procedure can enhance early detection, improve patient outcomes, and reduce the overall burden of pNTs in pediatric populations.

CIN is a hallmark of cancer characterized by an increased rate of chromosomal alterations, including gains, losses, and structural rearrangements. This instability leads to genetic diversity within tumor cell populations, driving tumorigenesis and contributing to cancer progression (60–62). Our data identified a number of common chromosomal alterations in ctDNA from patients with pNT and showed that copy-number alterations in specific chromosomal regions might serve as potential predictive markers for risk stratification in pNT. These segmental alterations identified in ctDNA, including gains of 17q and 7 and losses of 3p, 11q, and 1p, closely mirrored those known in pNT tumor tissue (1, 63). The copy-number alterations in specific chromosomal regions were highly concordant between the two, reinforcing the potential of ctDNA as a reliable, noninvasive tool for assessing CIN in pNT.

HRD refers to a cellular defect in the ability to repair DNA double-strand breaks through the homologous recombination repair (HRR) pathway, which can lead to genomic instability and an increased susceptibility to cancer (64). HRD is a therapeutic vulnerability in cancers, and HRD-positive tumors are more sensitive to PARPi or platinum-based neoadjuvant chemotherapy in breast and ovarian cancers (65–67). In this study, we found that HRD scores inferred from ctDNA in the high- and intermediate-risk groups were significantly higher compared with those in the low-/very low–risk group. This suggests that HRD may serve as a potential biomarker to guide therapeutic strategies for pNTs, such as the use of PARPi or platinum-based chemotherapy, in patients with elevated HRD scores. Although a few low- and intermediate-risk samples exhibited unexpectedly high HRD scores, these cases may reflect biological or technical outliers, possibly influenced by low ctDNA fractions or tumor heterogeneity. Nevertheless, these deviations did not affect the overall trend or statistical significance.

In summary, our data highlight the effectiveness of pNT-UCAD as a promising approach for diagnosis and risk stratification of pediatric neuroblastic tumors. Particularly, this method is minimally invasive, rapid, reliable, cost-effective, and does not rely on the detection of specific genomic alterations. Such minimally invasive approach also holds significant potential for real-time monitoring of tumor progression and treatment response, aiding in guiding personalized treatment strategies for patients with pNT. CNA can occur in other pediatric solid tumors, such as renal and hepatic malignancies, as well as sarcomas. Future studies involving larger, multi-cancer cohorts will be necessary to systematically characterize CNA landscapes across different pediatric tumor types. Such efforts will help to determine the specificity and diagnostic utility of LP-WGS in distinguishing pNTs from other childhood malignancies.

## Supplementary Material

Supplementary Table S1 Table S1. The demographic characteristics of patients.

Supplementary Table S2 Table S2. The characteristics of ctDNA samples from pNT patients and healthy controls.

Supplementary Table S3 Table S3. Copy-number Z-score of ctDNA samples from pNT patients and healthy controls.

## Data Availability

The LP-WGS data in this study were available from the Genome Sequence Archive (RRID: SCR_025826; ref. 68) in National Genomics Data Center (69), China National Center for Bioinformation/Beijing Institute of Genomics, and Chinese Academy of Sciences under accession number HRA009839.
